# The Regulatory Functions of the Multiple Alternative Sigma Factors RpoE, RpoHI, and RpoHII Depend on the Growth Phase in *Rhodobacter sphaeroides*

**DOI:** 10.3390/microorganisms11112678

**Published:** 2023-10-31

**Authors:** Jing Zhang, Meijia Zheng, Zizhong Tang, Shanpu Zhong, Tongliang Bu, Qingfeng Li

**Affiliations:** College of Life Sciences, Sichuan Agricultural University, Ya’an 625014, China; 18650193561@163.com (J.Z.); meijiazheng3426@163.com (M.Z.); 14126@sicau.edu.cn (Z.T.); heavenmile@163.com (S.Z.); tlbu@sicau.edu.cn (T.B.)

**Keywords:** *Rhodobacter sphaeroides*, growth phase-dependent, alternative sigma factor, RpoE, RpoHI, RpoHII

## Abstract

Bacterial growth, under laboratory conditions or in a natural environment, goes through different growth phases. Some gene expressions are regulated with respect to the growth phase, which allows bacteria to adapt to changing conditions. Among them, many gene transcriptions are controlled by RpoHI or RpoHII in *Rhodobacter sphaeroides*. In a previous study, it was proven that the alternative sigma factors, RpoE, RpoHI, and RpoHII, are the major regulators of oxidative stress. Moreover, the growth of bacteria reached a stationary phase, and following the outgrowth, *rpoE, rpoHI,* and *rpoHII* mRNAs increased with respect to the growth phase. In this study, we demonstrated the regulatory function of alternative sigma factors in the *rsp_0557* gene. The gene *rsp_0557* is expressed with respect to the growth phase and belongs to the RpoHI/RpoHII regulons. Reporter assays showed that the antisigma factor ChrR turns on or over the RpoE activity to regulate *rsp_0557* expression across the growth phase. In the exponential phase, RpoHII and sRNA Pos19 regulate the expression of *rsp_0557* to an appropriate level under RpoE control. In the stationary phase, RpoHI and Pos19 stabilize the transcription of *rsp_0557* at a high level. During outgrowth, RpoHI negatively regulates the transcription of *rsp_0557*. Taken together, our data indicate that these regulators are recruited by cells to adapt to or survive under different conditions throughout the growth phase. However, they still did not display all of the regulators involved in growth phase-dependent regulation. More research is still needed to learn more about the interaction between the regulators and the process of adapting to changed growth conditions and environments.

## 1. Introduction

Throughout different growth phases, bacteria are always exposed to varying environments that influence survival, biosynthesis, and metabolism, which are recognized as general stresses, including osmotic stress, extreme pH, oxidative stress, and nutrient deficiency. Several decades ago, some molecular mechanisms that enable bacteria to adapt or survive in similar stress situations were identified. The function of sigma factors in growth phase-dependent gene regulation has been elucidated, but the mechanisms are not clear. Nevertheless, other players in growth phase-dependent gene regulation have not been identified, such as small noncoding RNAs, small proteins, or dwarf signal molecules.

To date, some regulatory factors that regulate gene expression in a growth phase-dependent manner have been identified in Gammaproteobacteria. For example, the process of entering the stationary phase is orderly; in this process, diverse regulators are involved at different levels, extending transcriptional, translational, and post-translational controls in *Enteric bacilli*. The main regulator of this process is the sigma factor. In many Gram-negative bacteria, the sigma factor RpoS regulates observable physiological changes and plays a major role in resistance to diverse stresses [1]. During the stationary phase, increased *rpoS* transcription contributes to enhancing *rpoS* transcription, improving the efficiency of translation, and increasing protein stability [2]. Some regulatory factors affect *rpoS* transcription in this process, such as the membrane sensor kinase BarA [2], weak acids such as benzoate or propionate [3], phosphorylated ArcA [4], etc. The RNA chaperon Hfq and sRNAs (OxyS, RprA, and DsrA) participate in the regulation of RpoS translation [5,6,7]. Moreover, it was revealed that nucleoid-associated proteins like Lrp, IHF, and Fis regulate gene expressions in the stationary phase of intestinal bacteria [1,8,9]. In *Escherichia coli*, many genes induced in the stationary phase are regulated by Lrp, which in turn is regulated by ppGpp [10,11]. The involvement of sigma factors in growth-dependent gene expression was also reported for the Gram-positive *Bacillus subtilis*. Genes belonging to the SigW regulon are highly upregulated at the initiation of the late stationary phase [12]. Another global regulator in the stationary phase of *B. subtilis* and other Gram-positive bacteria is CodY. CodY represses more than one hundred genes during exponential growth. The activation of its repressor function is under the control of branched-chain amino acids and GTP [13], whereas the inactivation of the CodY repressor is mediated by (p)ppGpp [14].

*Rhodobacter sphaeroides* is a facultative phototrophic Alphaproteobacterium, which does not have a sigma factor with a function similar to that of RpoS in *E. coli* [15]. There are three alternative sigma factors in *R. sphaeroides*: RpoE, RpoHI, and RpoHII,. RpoHI and RpoHII are the main factors responsible for resistance to oxidative and heat stress responses and are involved in growth phase-dependent gene regulation [16]. The RpoHI and RpoHII regulons have considerable overlap; both of them and RpoE are considered to be global regulators in the oxidative stress response of *R. sphaeroides*, and RpoHII transcription is controlled by another sigma factor, RpoE [17,18,19]. The antisigma factor, ChrR, sequesters RpoE under normal conditions [20]. However, RpoE is activated after the degradation of ChrR under the action of the proteases DegS and RseP under oxidative stress. The process of degradation in response to singlet oxygen is promoted by the unknown functional proteins RSP_1090 and RSP_1091 [21]. RpoE, RpoHI, and RpoHII also regulate the expression of small noncoding RNAs, which play a role in the resistance to different stresses in *R. sphaeroides* [21,22,23,24,25]; e.g., noncoding small RNA Pos19 transcription is controlled by RpoE and increases under oxidative stress [26]. Moreover, the sRNA StsR is induced by iron starvation under the control of RpoHI/RpoHII [24,25]. In *R. sphaeroides*, the genes encoding the PhyR-NepR-σ^EcfG^ with a function in the general stress response also show different expressions across growth phases [27,28]. Recently, it was found that RNase E can affect bacterial adaptation to different growth conditions [29].

*R. sphaeroides* is a well-studied model organism for biological processes. In order to study more about the regulatory functions of RpoE, RpoHI, and RpoHII in gene expression depending on the growth phase in *R. sphaeroides*, we constructed a reporter plasmid that contained a promoter recognized by RpoHI/RpoHII, and measured the promoter activity across growth phases in various *R. sphaeroides* strains to understand the interplay between sigma factors and sRNA in different growth phases.

## 2. Materials and Methods

### 2.1. Plasmids, Strains, Media, and Culture Conditions

*Rhodobacter sphaeroides* 2.4.1 was the parental strain used to construct the mutant strains in this study. All strains are listed in Table 1. *R. sphaeroides* strains were cultivated in the dark at 32 °C on malate minimal-salt (RÄ) medium solid media containing 1.6% (*w*/*v*) agar or in liquid media [30]. For semi-aerobic growth conditions, the liquid medium was filled to 80% of the maximum volume of the Erlenmeyer flasks and shaken at 140 rpm. If necessary, gentamicin (10 μg mL^–1^), tetracycline (1.5 μg mL^–1^), kanamycin (25 μg mL^–1^), trimethoprim (200 μg mL^–1^), or spectinomycin (10 μg mL^–1^) was added to the media. *E. coli* strains were cultivated in Lysogeny Broth-Lennox (LB) media or on solid growth media containing 1.6% (*w*/*v*) agar, and cells were continuously shaken at 180 rpm at 37 °C. When necessary, ampicillin (200 μg mL^–1^), tetracycline (20 μg mL^–1^), or gentamicin (10 μg mL^–1^) was added to the growth media.

### 2.2. Expression and Purification of His_6_-Tagged RpoHI or RpoHII Protein

To produce 6 × His-tagged RpoHI or RpoHII protein, the region encoding *rpoHI* or *rpoHII* was amplified using the genomic DNA of *R. sphaeroides* 2.4.1 as a template. Primers used in PCR were (see Table 2) designed with NdeI and NotI restriction sites to amplify *rpoHI* (primers are RpoHI-E-f/r) or *rpoHII* (primers are RpoHII-E-f/r). The CloneJET PCR cloning kit (Thermo) was used to sub-clone PCR amplification products with vector pJET1.2. After digestion with NdeI and NotI, the *rpoHI* or *rpoHII* fragments were cloned into the expression vector pET30b to generate the RpoHI protein expression vector or RpoHII protein expression vector.

The resulting plasmid of the protein expression vector was transferred into *E. coli* BL21 (DE3) cells for RpoHI-His_6_ or RpoHII-His_6_ (N-terminal His_6_-tagged recombinant protein) expression. Recombinants were cultivated in LB medium at 37 °C with a final concentration of kanamycin (25 μg mL^−1^) until the optical density achieved 0.8–0.9 at 600 nm. Next, a final concentration of 1 mM IPTG was used to induce protein expressions, and the cells were harvested after 3 h (RpoHI protein) or 20 h (RpoHII protein) grown at 28 °C. Culture cell pellets were collected and resuspended in PBS buffer at 4 °C. Then, sonication was performed with an ultrasonic crusher on ice for 3 s with a 5 s interval break until the bacterial solution was clear. Cell debris and supernatant were separated using centrifugation at 8000 rpm at 4 °C for 10 min to obtain the recombinant protein. The recombinant protein samples were analyzed using a 12% SDS–polyacrylamide gel.

For the purification of RpoHI or RpoHII protein, the broken cell debris was purified using a His-Tagged Protein Purification Kit (Inclusion Body Protein, ComWin). The 12% SDS-PAGE was used to analyze the purified 6 × His-tagged proteins.

### 2.3. Electrophoresis Mobility Shift Assay

The primer set 0557-EMSA-F/R (Table 2) labeled with FAM was used to amplify the promoter fragment of *rsp_0557* from the genome of *R. sphaeroides* 2.4.1 via PCR. The 0557-M12-F/R primers and 0557-M35-F/R primers were used to amplify the *rsp_0557* promoter sequence after the mutation in the −12 and −35 regions. The fragment of *rsp_0557* promoter (273 bp) was incubated using purified RpoHI protein or RpoHII protein in the reaction buffer (0.75 M NaCl, 0.5 mM DTT, 50 mM Tris, 0.5 mM EDTA, pH 7.4) at 25 °C for 30 min. The reaction solution was then checked using 8% native PAGE in 0.5 × TBE electrophoresis buffer. A 282 bp fragment including the *rsp_0960* promoter was used as a negative control.

### 2.4. Construction of Reporter Plasmid

The fragment of the *rsp_0557* promoter was transcriptionally fused to the reporter gene *lacZ*. The primer set RSP_0557-f/r (Table 2) was used to amplify the promoter fragment of *rsp_0557* from the genome of *R. sphaeroides* 2.4.1 via PCR. The target fragment was sub-cloned using the vector pJET1.2 (Thermo), digested with the restriction enzymes HindIII and EcoRI, and ligated into EcoRI/HindIII-digested pBBRIMCS5-*lacZ* to generate the *rsp_0557* reporter plasmid vector pBBRI-*0557*-*lacZ* with the *lacZ* reporter gene (Table 1). The reporter plasmid was transferred into various strains, including *R. sphaeroides* wild type, ΔRpoHI (*rpoHI* deletion mutant), ΔRpoHII (*rpoHII* deletion mutant), ΔRpoHI/RpoHII (*rpoHI* and *rpoHII* double deletion mutant), ΔChrR (*chrR* deletion mutant), or TF18 (*rpoE*-*chrR* deletion mutant) by diparental conjugation.

### 2.5. Construction of pos19 Deletion Strain

Splicing by overlap extension (SOE) PCR was used to construct the *pos19* deletion strain (ΔPos19, Table 1) using the suicide vector pK18*mobsacB*::*pos19*. Primer pairs 0019-XbaI-up/0019-up or 0019-down/0019-BamHI-down were used to amplify an upstream or downstream fragment of *pos19* (Table 2) using the genomic DNA of *R. sphaeroides* 2.4.1 as a template. The upstream and downstream fragments were spliced by SOE PCR, which resulted in a 472 bp fragment. Following the XbaI/BamHI digestion, the fragment was ligated into the pK18*mobsacB* to generate pK18*mobsacB*::*pos19*.

For the construction of ΔPos19, the pK18*mobsacB*::*pos19* was conjugated to *R. sphaeroides* 2.4.1. After a single homologous recombination event, the kanamycin-resistant clones were screened using RÄ media with kanamycin and then cultivated in media without any antibiotics for about 60 h. Subsequently, the culture was spread on malate minimal salt plates at a concentration of 10% (*w*/*w*) sucrose to select for the deletion strain. Finally, potential mutants were checked via PCR using 0019-up-XbaI/0019-down-BamHI primers.

### 2.6. Overexpression Plasmid Construction

In order to construct the overexpression vector of *pos19* (pRKPos19, Table 1), the primers pPos19-F/R were used to amplify by PCR to obtain a 339 bp fragment without a native promoter (Table 2). The target fragment was sub-cloned by the vector pJET1.2 Blunt. The target fragment was ligated into the vector pRK16S after the degradation of BamHI and EcoRI restriction enzymes, which contained a 16S promoter.

### 2.7. Diparental Conjugation

The method of biparental conjugation was used as described in a previous study [31,32], with slight modifications in this study. *E. coli* S17-1 carried the corresponding plasmid to the *R. sphaeroides* strains. First, 0.5 mL of *E. coli* S17-1 carrying the plasmid and 1 mL of *R. sphaeroides* cells (OD_660_ = 0.6–0.8) were collected via centrifugation at 4000 rpm at 25 °C for 5 min. The 150 μL RÄ media was used to resuspend the cell pellet. Then, the mixed suspension was pipetted onto the Carl-Roth membrane (0.45 μm) and incubated on a PY agar plate at 32 °C. After 6 h of cultivation, the cells were washed from the membrane, streaked on RÄ agar plates containing the corresponding antibiotics, and cultivated at 32 °C for 3 days.

### 2.8. β-Galactosidase Assay

*R. sphaeroides* strains containing a plasmid-borne RSP_0557-*lacZ* fusion were cultivated in RÄ media under micro-aerobic growth conditions. Cells were harvested across growth phases after inoculation in RÄ liquid media from 0 h to 72 h and outgrowth (culture was diluted to fresh media with an optical density of 0.2 at 660 nm) at 24 h, 48 h, and 72 h of cultivation. Different volumes of samples were collected for testing according to the OD_660_ of the culture. The β-galactosidase activity of transcriptional fusions was measured using the hydrolysis of O-nitrophenyl-β-D-galactopyranoside (ONPG) and expressed as Miller Units [33]. At least three biological repeats were measured.

### 2.9. Quantitative Real-Time RT PCR

*R. sphaeroides* 2.4.1 cultures were harvested via centrifugation at 10,000 rpm for 10 min at 4 °C in different growth phases. Then, RNA was extracted and purified for RT-PCR using the RNAprep Pure Cell/Characteristic Kit of TIANGEN. As described in the manufacturer’s manual, reverse transcription and PCR reactions were performed using the Brilliant III Ultra-Fast SYBR Green QRT-PCR Master Mix (Agilent, Santa Clara, CA, USA), and amplification was performed using a CFX96 RT-PCR machine (Bio-Rad, Hercules, CA, USA). To ensure the accuracy of the data, all RT-PCR experiments were conducted in triplicate. The *rpoZ* gene was used as an endogenous control. Relative mRNA levels were calculated according to the Pfaffl method. The primers are listed in Table 2.

### 2.10. Data Analysis

Microsoft Office Professional Plus 2010 and GraphPad Prism 5 were employed for data and image processing, respectively.

## 3. Results

### 3.1. The rsp_0557 Is Regulated by RpoHI/RpoHII in R. sphaeroides

**Figure 1:**
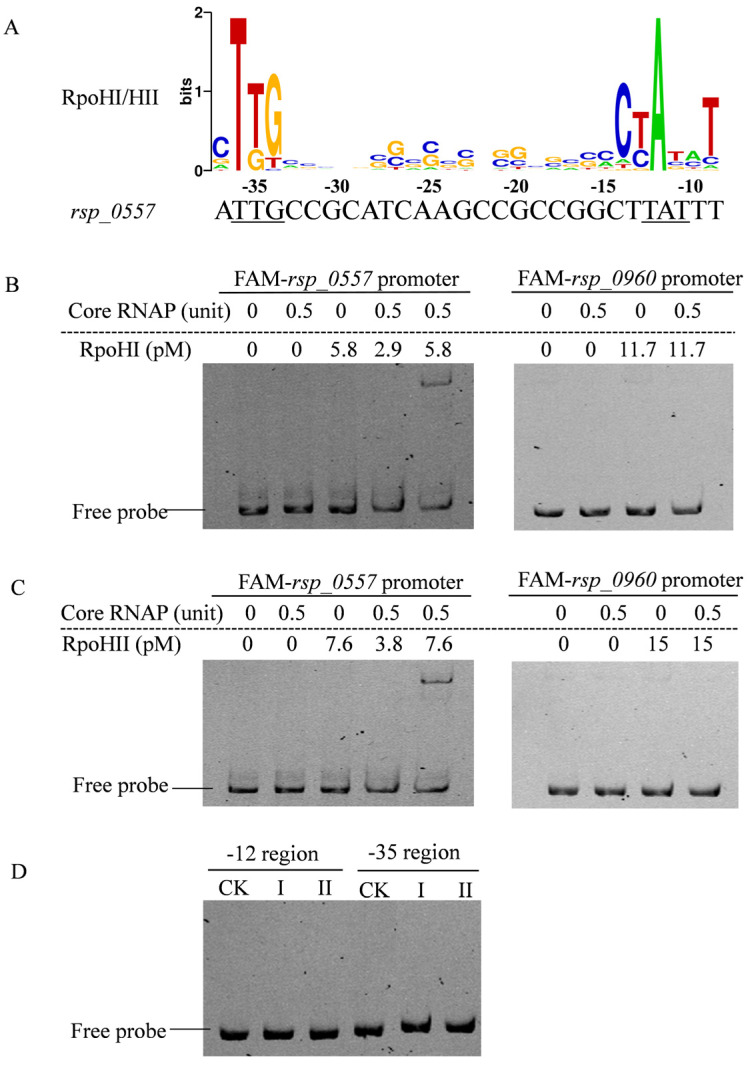
EMSAs of RpoHI or RpoHII interactions with the *rsp_0557* promoter. (**A**) The upstream region of *rsp_0557* matching the consensus sequences of RpoHI/HII promoter -35 (TTG) and -12 (TAT) using the MEME program. (**B**,**C**) A FAM-labeled 282 bp fragment of the *rsp_0557* promoter was mixed with a certain amount (unit) of *E. coli* core RNA polymerase (core RNAP) and a certain amount (pM) of purified RpoH protein with His tag, and then incubated at room temperature for 30 min. A FAM-labeled 273 bp fragment of the *rsp_ 0960* promoter used as a negative control. (**D**) The *rsp_0557* promoter mutated in -12 or -35 region was mixed with core RNAP and purified RpoH protein. CK is blank control, I represents RpoHI protein, and II represents RpoHII protein.

According to transcriptome data, most growth phase-dependent expression genes are regulated by alternative sigma factors, such as RpoHI, RpoHII, or either [16]. Within the group, we found that *rsp_0557* presents growth phase-dependent expression, which encodes a small protein with 70 amino acids containing a DUF1127 domain. RSP_0557 is homologous to RSP_6037, and the latter also has a DUF1127 domain that functions in RNA turnover and sRNA maturation [23,34]. Earlier research has demonstrated that the presence of photo-oxidative stress and hydrogen peroxide leads to an elevation in *rsp_0557* mRNA, which is influenced by RpoHI or RpoHII sigma factors in *R. sphaeroides* [35]. To verify the interactions between the promoter of *rsp_0557* and RpoHI or RpoHII, the region upstream of the transcription start site of the *rsp_0557* gene was searched for common motifs using the MEME program. We found that the TTG in the -35 region and TAT in the -12 region of the *rsp_0557* transcription start site corresponded to the conserved motif recognized by RpoHI/RpoHII (Figure 1B) [16].

To further demonstrate the interactions between the promoter of *rsp_0557* and RpoHI or RpoHII, electrophoresis mobility shift assays were carried out to determine whether RpoHI protein interacted with the *rsp_0557* promoter in vitro. The RpoHI protein at final concentrations of 2.9 pM and 5.8 pM was incubated with 160 ng *rsp_0557* promoter. As shown in Figure 1B, the RpoHI protein did not bind to the *rsp_0557* promoter without the RNA polymerase core enzyme; however, a shifted band was observed when the RNA polymerase core enzyme was present, which confirmed the interaction between the RpoHI protein and the *rsp_0557* promoter. No interaction was observed between RpoHI and the negative control of the *rsp_0960* promoter (Figure 1B). As shown in Figure 1C, we also proved that the RpoHII protein interacted directly with the promoter region of *rsp_0557* when the core enzyme was present, as well as with the RpoHI protein. In addition, after the TTG and TAT in the -35 and -12 regions of the *rsp_0557* transcription start site were mutated, the interaction was still not observed after adding excessive RpoHI or RpoHII protein under the action of the RNA polymerase core enzyme (Figure 1D). This suggests that the interaction of RpoHI or RpoHII with the *rsp_0557* promoter region was direct and specific.

### 3.2. Growth Phase-Dependent Expression of rsp_0557

To analyze *rsp_0557* expression with respect to growth phases, we tested the *rsp_0557* promoter transcriptional activity to indirectly reflect the expression level of *rsp_0557* using the reporter plasmid pBBRI-*0557*-*lacZ* in the wild-type strain. Cells from the reporter strain were harvested at various time intervals throughout the growth phases (0 h–72 h of growth) and outgrowth phases at 24 h, 48 h, and 72 h of cultivation under semiaerobic growth conditions.

**Figure 2:**
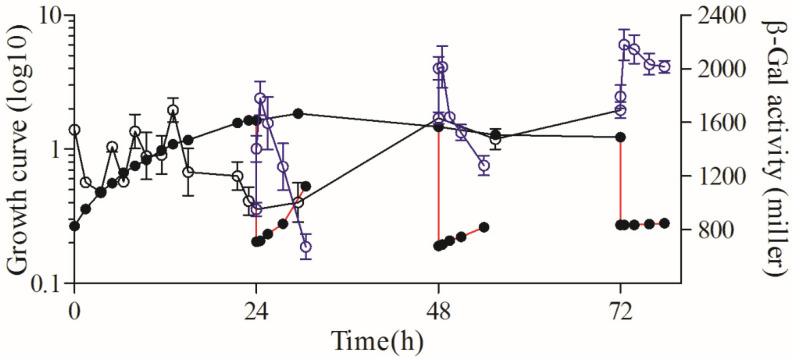
β-Gal activity throughout the growth phase in *R. sphaeroides* 2.4.1. The line connected by the solid circle represents the growth curve throughout the growth phase; the solid circle connected with the red line represents the growth curve of outgrowth; the line connected by the hollow circle represents the β-Gal activity throughout the growth phase; the blue hollow circle connected with the blue line represents the β-Gal activity of outgrowth.

The β-Gal results showed that the activity of the *rsp_0557* promoter changed continuously and significantly in different culture periods in the wild-type strain, particularly during the exponential phase and the early stationary phase (Figure 2), which indicates that *rsp_0557* is a growth phase-dependent expression gene. The activity of β-Gal was in a state of constant fluctuation before 12 h. Then, the activity decreased rapidly, with only 952 Miller Units at 24 h of growth. After 48 h, the β-Gal activity increased from the lowest activity at 24 h of growth to a high level. After cultures were diluted in fresh media to OD_660_ of 0.2 at 24 h, 48 h, and 72 h of growth, a temporary increase in the β-Gal activity was detected. Following further cultivation, the β-Gal activity declined steadily. These results further prove that *rsp_0557* exhibits growth phase-dependent expression.

### 3.3. Major Roles of RpoHI/RpoHII in the Transcription of rsp_0557 in Different Growth Phases

A recent study indicated that the regulons of RpoHI and RpoHII were partially overlapped [17]. Although RpoHI mainly plays a role after heat stress and photo-oxidative stress, RpoHII mainly functions under exposure to photo-oxidative stress [19]. RpoHII activates many genes with different functions in response to oxidative stress [17,18]. Moreover, other than heat shock and singlet oxygen, previous studies have shown that the RpoHI/RpoHII-dependent promoter can also be activated by several other stress factors, such as organic peroxide, hydrogen peroxide, superoxide, and CdCl_2_ [23]. The alternative sigma factors of RpoHI/HII regulate entry into the stationary phase and subsequent outgrowth [16].

To learn more about the effect of RpoHI/RpoHII on *rsp_0557* in different growth phases, we fused the reporter plasmid pBBRI-*0557*-*lacZ* into the *rpoHI* deletion mutant, along with *rpoHII* deletion mutant as well as *rpoHI*/*rpoHII* double deletion mutant.

**Figure 3:**
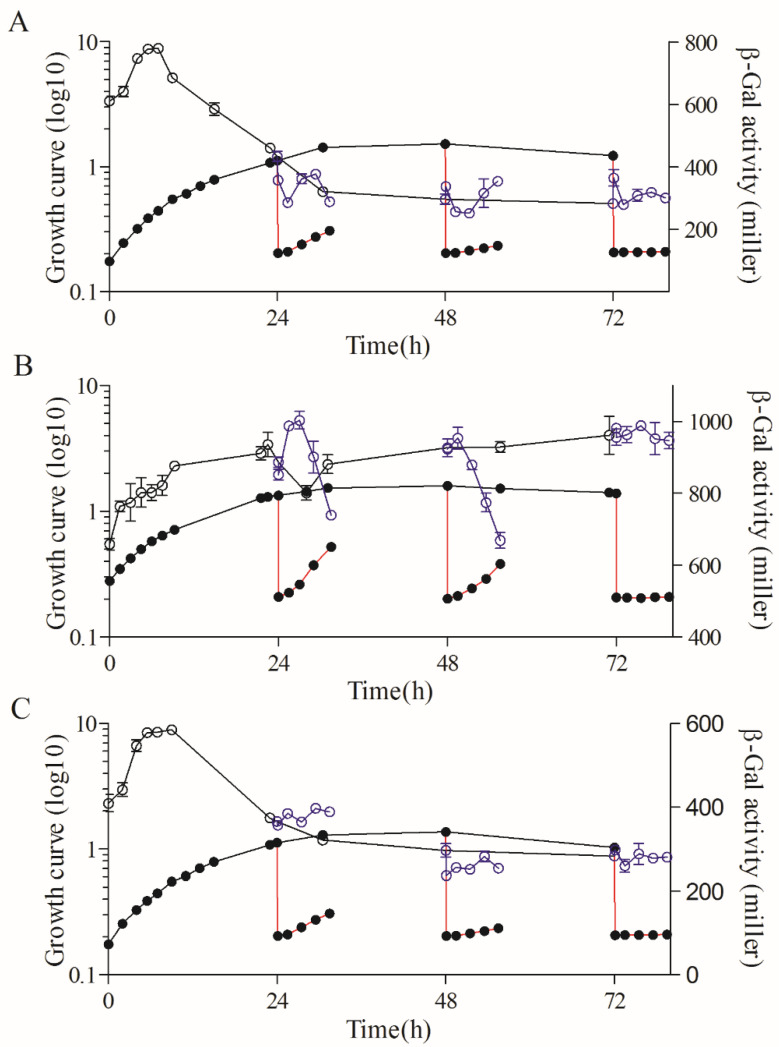
Major roles of RpoHI/RpoHII on the expression of *rsp_0557* during different growth phases. (**A**) *rpoHI* deletion mutant. (**B**) *rpoHII* deletion mutant. (**C**) *rpoHI/rpoHII* double deletion mutant. The line connected by the solid circle represents the growth curve throughout the growth phase; the solid circle connected with the red line represents the growth curve of outgrowth; the line connected by the hollow circle represents the β-Gal activity throughout the growth phase; the blue hollow circle connected with the blue line represents the β-Gal activity of outgrowth.

Compared to the wild type, the expression pattern of *rsp_0557* was completely different in the absence of *rpoHI* (Figure 2 and Figure 3A). The activity of β-Gal in the *rpoHI* deletion mutant was significantly lower than that in the wild-type strain. The transcriptional activity increased at the onset of the exponential phase and then decreased gradually, and was maintained at a low level when the culture entered the stationary phase in the *rpoHI* deletion mutant. The β-Gal activity remained almost unchanged, especially at 48 h and 72 h of outgrowth periods. The above shows that RpoHI mainly has a function in the outgrowth phase due to the duration of the stationary phase.

In the *rpoHII* deletion mutant, the promoter activity of *rsp_0557* was significantly higher than that in the *rpoHI* deletion mutant, but definitely lower than that in the wild type (Figure 2 and Figure 3A,B). The β-Gal activity was different during the whole growth phase. The β-Gal activity exhibited a consistent increase from the initial exponential phase to the initial stationary phase, followed by its maintenance in a high platform during the stationary phase in the *rpoHII* deletion mutant. The activity of β-Gal increased first and then decreased in the 24 h of outgrowth, and decreased continuously in the 48 h of outgrowth, similarly to the wild-type strain. However, it remained unchanged in the 72 h of outgrowth. This indicates that RpoHI and RpoHII regulate *rsp_0557* transcription in different strategies during outgrowth, depending on the duration of the stationary phase, but the role of RpoHI is greater.

**Figure 4:**
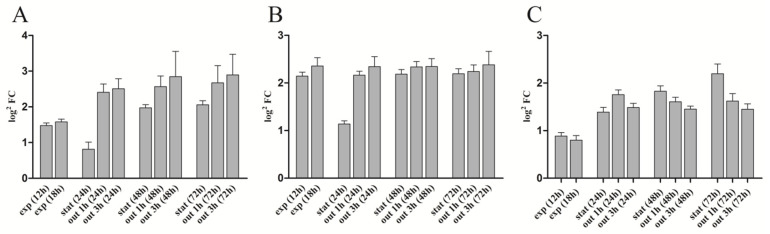
Real-time quantitative PCR results. The ratio of expression (log^2^-fold change) of selected genes as determined using real-time RT-PCR at different phases compared to the exponential phase of 6 h in the wild type of *R. sphaeroides*. The results were obtained from three independent biological experiments. The error bars represent the standard error of the mean. (**A**) The relative expression level of *rpoHI* in wild-type strain. (**B**) The relative expression level of *rpoHII* in wild-type strain. (**C**) The relative expression level of *rpoE* in wild-type strain.

The measurement of *rsp_0557* promoter activity across growth phases was not significantly different between the *rpoHI*/*HII* double deletion mutant and the *rpoHI* deletion mutant; the sole difference was that the activity of β-Gal in the *rpoHI* deletion mutant was higher before 24 h compared to the *rpoHI*/*HII* double deletion mutant (Figure 3A,C). During the outgrowth, the β-Gal activity remained constant. Because RpoHI and RpoHII regulated the outgrowth period of the stationary phase of *R. sphaeroides* [16], therefore, when both RpoHI and RpoHII were removed, the β-Gal activity during the outgrowth phase was almost unchanged. In addition, we also measured the relative expression levels of *rpoHI* and *rpoHII* in the wild-type strain using the 6 h expression level as a control (Figure 4A,B). The results showed that the expression level of *rpoHI* did not change significantly during the exponential phase. At 24 h of the stationary phase, the expression level of *rpoHI* significantly decreased compared to the exponential phase. However, at 48 h of the stationary phase, the expression level increased compared to that at 24 h, and at 72 h of the stationary phase, there was no significant change compared to 48 h. During the three outgrowth phases of the stationary phase, the expression level of *rpoHI* increased when the culture was diluted (Figure 4A). The *rpoHII* expression results showed that the trend of *rpoHII* expression was similar to that of *rpoHI* during the exponential and stationary phase; however, the relative expression level of *rpoHII* was higher during the exponential phase of 12 and 18 h compared to that of *rpoHII*. In the outgrowth phase at 24 h, the expression of *rpoHII* significantly increased after diluting the culture. During the 48 and 72 h outgrowth phases, there was no significant change in *rpoHII* expression after diluting the culture (Figure 4B). The above results indicate that RpoHI and RpoHII are important regulators of gene expression during the outgrowth phase. RpoHI produces a marked effect in the stationary and outgrowth phases. RpoHII mainly affected the lag phase, exponential phase, and transition phases from the late exponential to the stationary phase.

### 3.4. Major Role of RpoE in the Transcription of rsp_0557 in Different Growth Phases

It has been revealed that the sigma factor RpoE has a central function in resistance to the photo-oxidative stress in *R. sphaeroides* [17,18,19]. RpoE regulates the transcription of its own gene and promotes the transcription of other genes, including photolyase genes, *rpoHII,* and some sRNAs [19]. The gene *rsp_0557* transcription is mediated by RpoHII, so we speculate that RpoE may indirectly influence the expression of *rsp_0557*. In order to determine the regulatory function of RpoE on *rsp_0557* expression in different growth phases, β-Gal activity was also assessed in the absence of RpoE (in the TF18 strain, *rpoE-chrR* deletion mutant).

**Figure 5:**
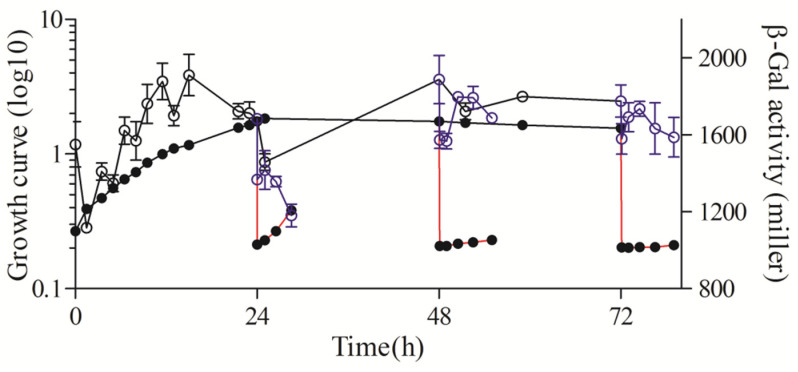
Growth phase-dependent expression of *rsp_0557* in *rpoE-chrR* deletion strain. The line connected by the solid circle represents the growth curve throughout the growth phase; the solid circle connected with the red line represents the growth curve of outgrowth; the line connected by the hollow circle represents the β-Gal activity throughout the growth phase; the blue hollow circle connected with the blue line represents the β-Gal activity of outgrowth.

During the whole growth phase, the pattern of *rsp_0557* expression in TF18 (Figure 5) was slightly different from that in the wild-type strain (Figure 2), especially during the exponential phase, early stationary phase, and outgrowth phase. In the exponential phase and early stationary phase, the β-Gal activity of the TF18 strain was higher than that of the wild-type strain. In the wild type, the β-Gal activity exhibited a rapid increase, followed by a subsequent decrease following culture dilution during the outgrowth phase. On the contrary, the activity directly decreased in the TF18 strain. Therefore, we predicted that RpoE has functions of both positive regulation and negative regulation of the expression of *rsp_0557*, which was used to balance the total number of the *rsp_0557* mRNA. Interestingly, if RpoE regulates the *rsp_0557* expression only via RpoHII, the expression of *rsp_0557* in the absence of RpoE across growth phases should be the same as that in the *rpoHII* deletion strain. However, the β-Gal activity in the TF18 strain was significantly higher than that in the *rpoHII* deletion strain (Figure 3 and Figure 5). In addition, we also measured the relative expression level of *rpoE* in the wild-type strain (Figure 4C), and the results showed that there was no significant change in the expression level of *rpoE* during the exponential phase. The *rpoE* expression level rose steadily over time during the stationary phase. During the 24 h outgrowth phase, when the culture was diluted, the expression level of *rpoE* first increased and then decreased. However, during the 48 h and 72 h outgrowth phases, the expression level of *rpoE* after dilution decreased. This indicates that RpoE regulates the expression of *rsp_0557* via RpoHII, as well as other regulatory factors.

### 3.5. sRNA Pos19 Is Involved in Regulating the Expression of rsp_0557

Based on the aforementioned research, we can conclude that RpoHI and RpoHII promote the expression of *rsp_0557*, and RpoE controls other regulators to inhibit the expression of *rsp_0557*, especially in the exponential and early stationary phases. Previous studies indicated that sRNA Pos19 is a negative regulator of *rsp_0557* expression. Pos19 has an RpoE-dependent promoter with a noncoding function that regulates the balance of thio-metabolism [26]. The overexpression of *pos19* reduces the content of glutathione in the exponential phase in cells, and the level of reactive oxygen species (ROS) does not change significantly, but the level of ROS in the exponential phase will increase after the removal of *pos19* [26]. According to microarray data analysis, overexpression of *pos19* significantly reduces the level of *rsp_0557* mRNA under singlet oxygen stress [26], and it has been shown that the sRNA Pos19 interacts with *rsp_0557* mRNA.

**Figure 6:**
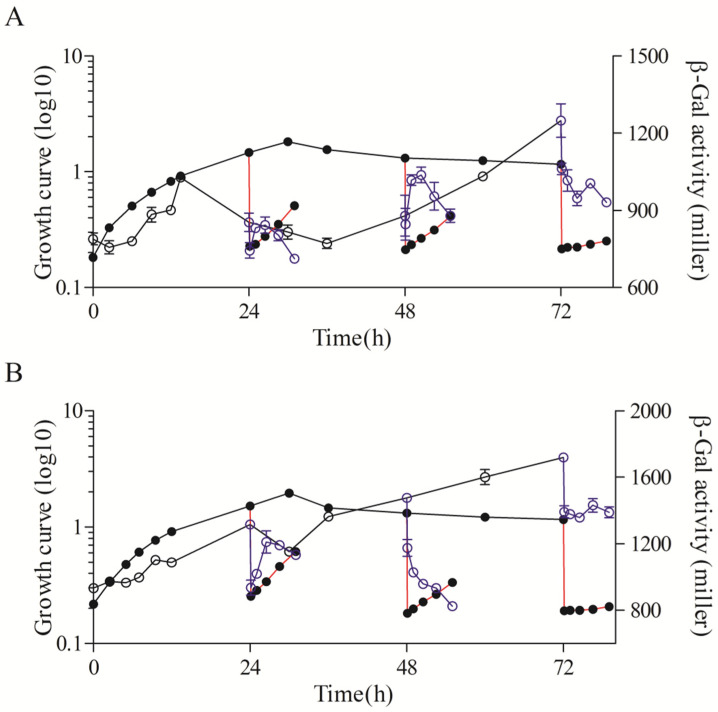
The major role of sRNA Pos19 on *rsp_0557* with respect to the growth phase. (**A**) *pos19* deletion strain. (**B**) *pos19* overexpression strain. The line connected by the solid circle represents the growth curve throughout the growth phase; the solid circle connected with the red line represents the growth curve of outgrowth; the line connected by the hollow circle represents the β-Gal activity throughout the growth phase; the blue hollow circle connected with the blue line represents the β-Gal activity of outgrowth.

**Figure 7:**
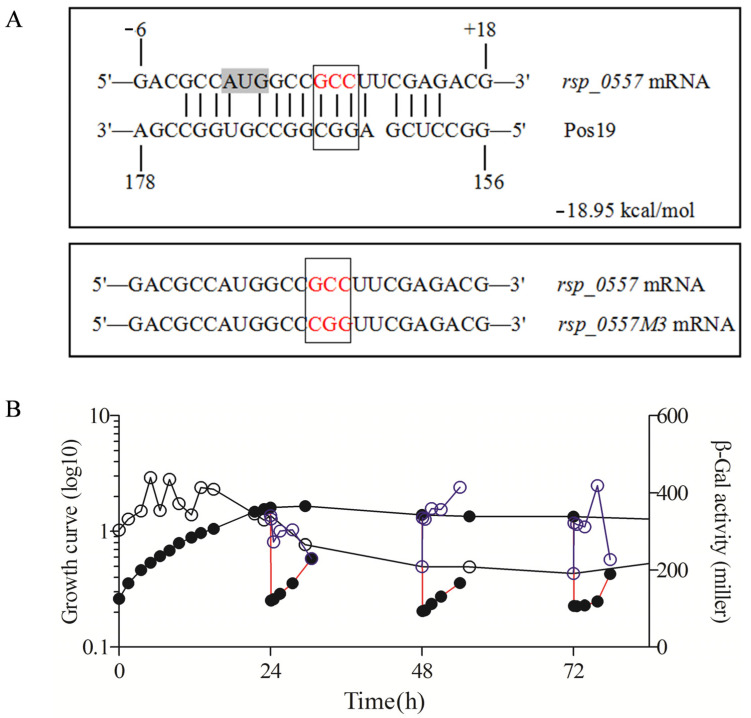
Transcriptional activity of the mutated *rsp_0557* promoter throughout the growth phase in the wild type. The GCC of 7-9 base sites relative to the start codon of *rsp_0557* mRNA was mutated to CGG. (**A**) Predicted interaction (seed region) between Pos19 and the *rsp_0557* mRNA using IntaRNA. The numbers on the *rsp_0557* mRNA sequence represent the position referred to by the start codon, while the numbers on Pos19 are relative to the 5′ end. The red text in the box represents the *rsp_0557* mRNA mutation site. (**B**) Mutated *rsp_0557* promoter sequences were inserted into pBBRIMCS5 fused with the *lacZ* gene. The resulting reporter plasmids were transferred to the wild type. Samples were collected for β-Gal assays. The line connected by the solid circle represents the growth curve throughout the growth phase; the solid circle connected with the red line represents the growth curve of outgrowth; the line connected by the hollow circle represents the β-Gal activity throughout the growth phase; the blue hollow circle connected with the blue line represents the β-Gal activity of outgrowth.

Thus, to determine the regulatory role of sRNA Pos19 in the expression of *rsp_0557*, we constructed a *pos19* deletion strain without a resistance marker. In contrast, the expression level of *rsp_0557* was lower in the *pos19* deletion mutant compared to the wild-type strain throughout the growth phases (Figure 2 and Figure 6A), which is inconsistent with our expectation that sRNA Pos19 inhibits the expression of *rsp_0557*. Therefore, to learn more about the influence of Pos19 on the expression of *rsp_0557*, we constructed a *pos19* overexpression strain with a 16S promoter. Compared with the wild-type strain, the overexpression of *pos19* significantly reduced the expression of *rsp_0557* (Figure 2 and Figure 6B) in the exponential phase and early stationary phase. The above experiments show that the deletion or overexpression of *pos19* will impede the transcription of *rsp_0557*. Therefore, in order to better understand the regulatory function of sRNA Pos19 in *rsp_0557*, the seed region (Figure 7A) was predicted using IntaRNA. We first analyzed the importance of the predicted binding region for *rsp_0557* promoter activity. After removing the 1-15 base of the *rsp_0557* mRNA seed region (Figure 7A), the β-Gal activity exhibited a remarkably diminished level (less than 50 Miller Units), which proves that the predicted binding region is necessary for the normal expression of *rsp_0557*. Then, we mutated the GCC of 7-9 base sites in the predicted *rsp_0557* mRNA seed region to CGG, namely *rsp_0557M3* (Figure 7A), and the expression pattern of *rsp_0557M3* was analyzed. The results showed that compared with the unmutated reporter (Figure 2), the mutated *rsp_0557* reporter plasmid resulted in less β-Gal activity than overall (Figure 7B), indicating that the GCC of 7-9 base sites in the seed region of *rsp_0557* mRNA is necessary for the normal expression of *rsp_0557*. Müller proved that after the 7-9 base complementary mutation of Pos19 and *rsp_0557* mRNA, there is no significant change in the β-Gal activity compared with the control [35], indicating that the combination of sRNA Pos19 and *rsp_0557* mRNA can better maintain the stability of *rsp_0557* mRNA, but the overexpression of *pos19* also inhibits the expression of *rsp_0557* in the exponential phase and early stationary phase.

### 3.6. ChrR Participates in Maintaining the Stability of rsp_0557 Expression Level

**Figure 8:**
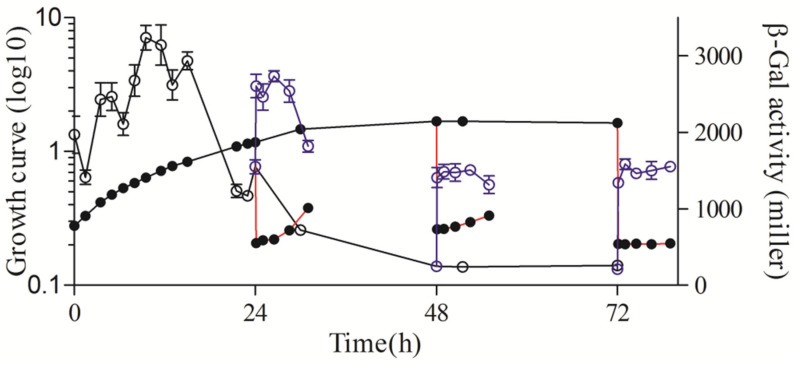
Growth phase-dependent expression of *rsp_0557* gene in *chrR* deletion strain. The line connected by the solid circle represents the growth curve throughout the growth phase; The solid circle connected with the red line represents the growth curve of outgrowth; The line connected by the hollow circle represents the β-Gal activity throughout the growth phase; The blue hollow circle connected with the blue line represents the β-Gal activity of outgrowth.

Under non-stress conditions, ChrR interacts with RpoE to form a heterodimer complex, thereby inhibiting the activity of RpoE protein. RpoE is released after the degradation of protein ChrR by proteases DegS and RseP under oxidative stress [21]. Therefore, RpoE remains active continuously after the deletion of ChrR, and RpoE actively and automatically regulates the expression of its own gene and activates the transcription of other target genes, including *rpoHII* [21]. Hence, for the purpose of understanding the regulatory function of ChrR in the *rsp_0557* expression, the β-Gal activity in the *chrR* deletion mutant was detected. The results showed that the *rsp_0557* expression level in the *chrR* deletion strain was significantly different from the other strains (Figure 2, Figure 3, Figure 4, Figure 5, Figure 6, Figure 7 and Figure 8). The β-Gal activity increased in the exponential phase more rapidly in the *chrR* deletion strain than in other strains (Figure 8), and then the level decreased rapidly from the exponential phase to the stationary phase. In addition, the β-Gal activity quickly increased and remained at a constant level after dilution at 24 h, 48 h, and 72 h of growth. It shows that the overexpression of RpoE will significantly increase the expression of *rsp_0557* during the exponential phase and inhibit the expression of *rsp_0557* during the stationary phase. Therefore, when RpoE is present, ChrR must exist simultaneously to maintain the normal expression level of *rsp_0557* in the stationary phase.

## 4. Discussion

Adapting and surviving under changing environments through the growth phase requires organisms to alter their gene expression pattern. In bacteria, the processes are mainly regulated by alternative sigma factors, which can combine with RNA polymerase to recognize specific promoters in cells and quickly adjust gene expressions in response to various signal stimuli. Furthermore, additional levels of regulation, post-transcriptional regulation, and translational regulation have been revealed [36]. To date, the molecular mechanisms of gene regulation with respect to growth phases are only known in a few species. For instance, RpoS, an alternative sigma factor regulates the process of entering the stationary phase, which alters the gene expression pattern so as to generate more resistant cells in enteric bacilli [1]. Previous studies verified that it is a complex mechanism of RpoS regulation, including transcriptional regulation, post-transcriptional regulation, and translational regulation. All these closely cooperate to resist several stresses [2]. These stresses trigger RpoS synthesis to increase the RpoS level in the stationary phase, enhance *rpoS* transcription, and improve protein stability and efficiency of translation [2,5,37]. During the stationary phase, RpoS modulates the expression of 10% of the genes, enhancing cellular resistance and adaptability to diverse stress scenarios in *E. coli* [38].

Alphaproteobacteria have an important role to play in animal and plant pathogens and symbionts, nitrogen fixation, carbon dioxide fixation, chemical product synthesis, and so on. The knowledge of gene regulation with respect to the growth phase is limited in Alphaproteobacteria. There is no RpoS homolog with regulatory function in the process of entry into the stationary phase, similar to enteric bacteria in Alphaproteobacteria, instead of other alternative sigma factors RpoHI/HII. As an illustration, a large number of genes showed altered mRNA levels in the stationary phase and following outgrowth in *R. sphaeroides*. RpoHI or RpoHII governs the transcription of numerous genes in *R. sphaeroides*, which react to diverse stresses [16]. The regulatory mechanisms of RpoHI/HII in growth phase gene regulation are not clear, but their functions in resistance to stresses in *R. sphaeroides* are well studied.

Our findings indicate that RpoHI/HII plays distinct roles in gene regulation during the growth phase. RpoHI and RpoHII control the expression of *rsp_0557* in response to singlet oxygen and heat shock, whereas RSP_0557 balances GSH metabolism [26]. However, RpoHI and RpoHII regulate the transcription of *rsp_0557* during distinct growth stages. We found that RpoHI enhanced the transcription of *rsp_0557* in the stationary phase rather than in the exponential phase (Figure 3A). After a prolonged stationary phase, the level of *rsp_0557* expression is decreased in outgrowth in the wild-type strain due to adaptation to changing environments. Nevertheless, cells lacking RpoHI have many defects, particularly the resistance to oxidative stress, and loss of the regulatory function on *rsp_0557* in the outgrowth phase after prolonged stationary phase (Figure 3A,C). The report assays in the RpoHII deletion mutant support that RpoHII directs the *rsp_0557* expression in the exponential phase; thus, RpoHII is the main regulator that adjusts *rsp_0557* expression in response to slightly changing environments (Figure 3B). Based on the quantitative results, we found that during the stationary phase and following outgrowth, RpoHI is more important than RpoHII to program gene expression for cell survival and adaptation to harsh living conditions. We can conclude that cells need to coordinate RpoHI and RpoHII levels to regulate the transcription of *rsp_0557* in *R. sphaeroides*, and RpoHI plays a dominant role. These regulatory mechanisms apparently differ from those of *E. coli*.

The key to the success of this regulation strategy is to control the activity of RpoHI and RpoHII and to ensure that the sigma factors are turned on or off in the right ways at the appropriate times. Under typical circumstances, the operations of alternative sigma factors are managed at various levels, such as transcription, post-transcription, and translation, which are intricate. It has been revealed that RpoE promotes the transcription of the *rpoHII* gene, whereas RpoHII controls the *rsp_0557* transcription. We can legitimately anticipate that the expression pattern of *rsp_0557* throughout the growth phase in the absence of RpoE should be similar to that in the *rpoHII* deletion strain, but we did not observe a promising result. In addition, the relative expression patterns of *rpoHII* and *rpoE* are also different (Figure 4B,C). Thus, it can be seen there are other regulators involved in the regulatory pathway of the *rsp_0557* expression. A previous study showed that an sRNA Pos19 negatively regulated the expression of *rsp_0557* at the post-transcriptional level in response to singlet oxygen and hydrogen peroxide. The transcription of Pos19 is controlled by RpoE as well as the *rpoHII* gene [26]. The deletion or overexpression strain showed that Pos19 not only decreased the transcription of *rsp_0557* in the exponential phase but also increased the *rsp_0557* level in the stationary phase. Therefore, we conclude that RpoE can affect the expression of *rsp_0557* via both the negative regulator Pos19 and positive regulation RpoHII, thereby controlling the amount of *rsp_0557* mRNA. Moreover, the RpoE overexpression strain (*chrR* deletion) indicated that RpoE activity mainly affects the stationary phase, in which the transcription of *rsp_0557* was at an extremely low level. The high expression level in the exponential phase suggests that the higher resistance of the chrR deletion strain compared to that of the wild-type strain leads to significantly reduced transcription. Therefore, the regulation of the underlying mechanisms in the stationary phase is more complex than our anticipation in the past. Hence, if degradation of ChrR is not the sole way to determine the total activity of RpoE during growth, it does so through an unknown pathway. We anticipated that ChrR would act as a regulator of RpoE activity in order to maintain equilibrium between the transcripts of Pos19 and rpoHII. The low expression of *rsp_0557* might be due to a lack of *chrR*, which causes the overexpression of *pos19* and *rpoHII* to break the balance of regulation. In addition, we can exclude the regulatory function of RpoE-RpoHII-StsR in response to stress during the stationary phase [36]. However, we still came to the conclusion that the expression of *rsp_0557* is regulated by RpoE via RpoHII and Pos19 to balance the GSH content in response to stress throughout the growth phase.

## 5. Conclusions

For now, it is impossible to show the regulatory functions of RpoHI and RpoHII on the expression of each gene during different growth phases in cells. Although the functions of RpoE, RpoHI, RpoHII, ChrR, and Pos19 have been identified in numerous studies, the intricate network that regulates growth phase-dependent genes remains a mystery. Various combinations of these regulators are recruited by cells to adapt to or survive under different conditions throughout the growth phase. To summarize, we suggest that ChrR activates RpoE activity in order to control gene expression throughout the growth stage. In the exponential phase, RpoHII and Pos19 regulate the expression of *rsp_0557* at an appropriate level under RpoE control. In the stationary phase, RpoHI and Pos19 stabilize the transcription of *rsp_0557* at high levels. In the outgrowth phase, RpoHI negatively regulates the transcription of *rsp_0557*. Our study demonstrates that the regulation mechanism across the growth phase is more complex than that in oxidative stress. It still did not display all of the regulators involved in growth phase-dependent regulation. Consequently, additional research is required to comprehend the various regulators governing these intricate networks, gain further insights into their interplay, and comprehend the regulation of adaptation to the changing conditions and growth phases.

## Figures and Tables

**Table 1:** Bacterial strains and plasmids used in this study.

Strain or Plasmid	Description or Relevant Features	Source/Reference
*E. coli* strains		
DH5α	For Sub-Cloning	Weidi
BL21 (DE3)	For protein expression	TIANGEN
S17-1	For diparental conjugation	Lab preservation
*R. sphaeroide* strains		
2.4.1	Wild type	Lab preservation
TF18	*rpoE chrR* mutation inWT, Tp^r^	Lab preservation
Δ*rpoHI*	WT *rpoHI*::Km^r^ cassette	Lab preservation
Δ*rpoHII*	WT *rpoHII*::Sp^r^ cassette	Lab preservation
Δ*rpoHI*Δ*rpoHII*	WT *rpoHIIrpoHI*::Km^r^.Sp^r^ cassette	Lab preservation
ΔChrR	WT *chrR*::Tp^r^ cassette	Lab preservation
ΔPos19	*Pos19* markerless deletion mutant	This study
Plasmids		
pJET1.2/blunt cloning vector	Ap^r^, 2.97 kb	Thermo
pBBRIMCS5-*lacZ*	Gm^r^	Lab preservation
pBBRI-*0557*-*lacZ*	Expression of *rsp_0557*; Gm^r^	This study
pK18*mobsacB*	suicide vector, *sacB* (sucrose sensitivity), Km^r^	Lab preservation
pK18*mobsacB*::*pos19*	For *pos19* deletion in 2.4.1	This study
pET30b	For protein expression	Lab preservation
pET30-RpoHI	RpoHI protein expression vector	This study
pET30-RpoHII	RpoHII protein expression vector	This study
pRK16S	Overexpression vector controlled by 16S promoter	Lab preservation
pRKPos19	*pos19* overexpression vector	This study

**Table 2:** Oligodeoxynucleotides used in this study.

Name	Sequence 5′-3′	Purpose
RpoHI-E-f	CATATGAGCACTTACACCAGCCTTCCC	EMSA
RpoHI-E-r	GCGGCCGCGGCGGGGATCGTCATG	EMSA
RpoHII-E-f	CATATGGCACTGGACGGATATACCGATC	EMSA
RpoHII-E-r	GCGGCCGCTAGGAGGAAGTGATGCACCTCC	EMSA
0557-EMSA-F	TGCCTGCAGGTCGACGATCCGCACGGCGCCAC	EMSA
0557-EMSA-R	CTCGAAGGCGGCCATGG	EMSA
0557-35M-F	GAACCAAGTTCCGACATCAAGC	EMSA
0557-35M-R	GTCGGAACTTGGTTCCGGA	EMSA
0557-12M-F	GCCGGCTAAATTGCCTGTTC	EMSA
0557-12M-R	CGTCTGAACAGGCAATTTAGCC	EMSA
0960-EMSA-F	TGCCTGCAGGTCGACGATTCCGACGGAAAACAGGATCC	EMSA
0960-EMSA-R	GCTTGCCTCCTGTAGGGCC	EMSA
FAM Fluorescent primer	TGCCTGCAGGTCGACGAT	EMSA
RSP_0557-f	GAATTCCCGCACGGCG	Cloning
RSP_0557-r	AAGCTTCTCGAAGGCGGC	Cloning
0019-XbaI-up	TCTAGACGATCAGGCGACGG	Cloning-knockout
0019-up	GGCCTTCTCCGGGGCACAGCTTACGCAGGGTCG	Cloning-knockout
0019-down	GACCCTGCGTAAGCTGTGCCCCGGAGAAGGCCC	Cloning-knockout
0019-BamHI-down	GGATCCTGCGCCCTCAG	Cloning-knockout
pPos19-F	GGATCCCGATCAACCCAAGCAGAA	Overexpression
pPos19-R	GAATTCGGATGTCCCGCTCAGG	Overexpression
rpoZ_RT-f	ATCGCGGAAGAGACCCAGAG	RT-PCR
rpoZ_RT-r	GAGCAGCGCCATCTGATCCT	RT-PCR
rpoHI_RT-f	GATCGCCAAGGATCT	RT-PCR
rpoHI_RT-r	CTGGTCGCTGTCTTCA	RT-PCR
rpoHII_RT-f	GCCGATGAACGACCTGAT	RT-PCR
rpoHII_RT-r	AAGAACAGCGCCTTCTGG	RT-PCR
rpoE_RT-f	GTCTGGCAGAAGGCTCAT	RT-PCR
rpoE_RT-r	GTTCTCCTGCTGCATCTC	RT-PCR

The restriction enzyme cleavage sites are indicated by underlined bases.

## Data Availability

All data are provided in full in this paper.
